# *In vivo* imaging of axonal transport in murine motor and sensory neurons

**DOI:** 10.1016/j.jneumeth.2015.09.018

**Published:** 2016-01-15

**Authors:** Katherine L. Gibbs, Bernadett Kalmar, James N. Sleigh, Linda Greensmith, Giampietro Schiavo

**Affiliations:** a Sobell Department of Motor Neuroscience & Movement Disorders, UCL Institute of Neurology, University College London, Queen Square, London WC1 N 3BG, UK; b Nuffield Department of Clinical Neurosciences, University of Oxford, John Radcliffe Hospital, Oxford OX3 9DU, UK

**Keywords:** Axonal transport, *In vivo*, Motor neurons, Sensory neurons, Neurological disease, Neurodegeneration

## Abstract

**Background:**

Axonal transport is essential for neuronal function and survival. Defects in axonal transport have been identified as an early pathological feature in several disorders of the nervous system. The visualisation and quantitative analysis of axonal transport *in vivo* in rodent models of neurological disease is therefore crucial to improve our understanding of disease pathogenesis and for the identification of novel therapeutics.

**New method:**

Here, we describe a method for the *in vivo* imaging of axonal transport of signalling endosomes in the sciatic nerve of live, anaesthetised mice*.*

**Results:**

This method allows the multiparametric, quantitative analysis of *in vivo* axonal transport in motor and sensory neurons of adult mice in control conditions and during disease progression.

**Comparison with existing methods:**

Previous *in vivo* imaging of the axonal transport of signalling endosomes has been limited to studies in nerve explant preparations or non-invasive approaches using magnetic resonance imaging; techniques that are hampered by major drawbacks such as tissue damage and low temporal and spatial resolution. This new method allows live imaging of the axonal transport of single endosomes in the sciatic nerve *in situ* and a more sensitive analysis of axonal transport kinetics than previous approaches.

**Conclusions:**

The method described in this paper allows an in-depth analysis of the characteristics of axonal transport in both motor and sensory neurons *in vivo.* It enables the detailed study of alterations in axonal transport in rodent models of neurological diseases and can be used to identify novel pharmacological modifiers of axonal transport*.*

## Highlights

• *In vivo* imaging of axonal transport in the sciatic nerve of live anesthetised mice.
• Signalling endosomes are monitored using fluorescent probes and confocal microscopy.
• Axonal transport in motor and sensory neurons can be differentiated.
• This method can be easily adapted to study the axonal transport of other cargoes.
• Potential use of this *in vivo* imaging approach in drug screening.

## Graphical abstract

**Figure:**
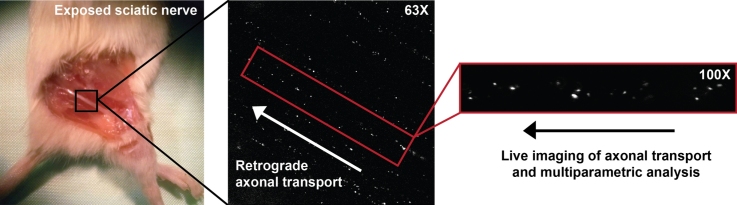

## 1 Introduction

Neurons are highly polarised cells with axons that can extend over a meter away from the cell body in large mammals. This unique morphology makes neurons particularly reliant on active intracellular transport. Long distance ATP-dependent transport along the axon is predominantly mediated by two families of microtubule-associated motor proteins: kinesins and cytoplasmic dynein (Maday et al., 2014). Axonal transport plays two main roles in neurons: (1) Supply and clearance: delivery of organelles and newly synthesised proteins, lipids and RNA to the axon and distal synapses, and removal of faulty ones to the soma for degradation and/or recycling; (2) Long-distance communication between the axon tip and the soma, including the transport of neurotrophic factors and their activated receptor complexes, which are essential for survival (Chowdary et al., 2012). It is therefore not surprising that alterations in axonal transport can be deleterious to neuronal function and survival, and that such deficits are proposed to be involved in the pathogenesis of several neurological diseases, including amyotrophic lateral sclerosis (ALS) (Bilsland et al., 2010, Kieran et al., 2005), Charcot Marie Tooth type 2 (d’Ydewalle et al., 2011), hereditary spastic paraplegia (Kasher et al., 2009) and diabetic neuropathy (Tomlinson and Mayer, 1984).

Due to the well-documented relationship between mitochondrial defects and neurodegenerative disease (Johri and Beal, 2012), live imaging of axonal transport *in vivo* has been previously described for mitochondria (Bilsland et al., 2010, Bolea et al., 2014, Misgeld et al., 2007). However, it is also apparent that an impairment of mitochondrial dynamics is not *per se* necessary or sufficient to trigger neuronal death in all neurodegenerative diseases. For example, it has been shown that reversing mitochondrial trafficking deficits does not affect neuronal death and disease progression in a mouse model of ALS (Zhu and Sheng, 2011). In addition, the axonal transport of several other cargoes has been reported to be affected in neurological diseases, including signalling endosomes, autophagosomes, RNA and lysosomes (Millecamps and Julien, 2013). Importantly, the axonal transport of these cargoes has been shown to be distinct from that of mitochondria and differentially regulated (Gibbs et al., 2015, Zala et al., 2013), highlighting the importance of studying their *in vivo* axonal transport in detail.

Past *in vivo* imaging of the axonal transport of signalling endosomes has been limited to non-invasive approaches using magnetic resonance imaging (Jouroukhin et al., 2013) or whole body fluorescence imaging (Schellingerhout et al., 2009). Such techniques do not allow for the analysis of axonal transport in specific neuronal types, nor permit the real-time visualisation of individual axons and endosomes. These shortcomings greatly limit the quantitative analysis of *in vivo* axonal transport and the use of these approaches for the evaluation of new therapeutic agents aimed at normalising axonal transport in disease models.

Here, we describe the *in vivo* imaging of the axonal transport of single endosomes in motor and sensory neurons of the sciatic nerve in live anaesthetised adult mice. Labelling of endosomes is achieved using one of two fluorescently tagged probes—the atoxic binding fragment of tetanus neurotoxin (H_C_T) (Bercsenyi et al., 2014, Bilsland et al., 2010, Bohnert and Schiavo, 2005, Deinhardt et al., 2006) or an antibody directed against the extracellular domain of the p75 neurotrophin receptor (α-p75^NTR^) (Deinhardt et al., 2007). α-p75^NTR^ allows the labelling of endosomes within p75^NTR^-expressing cells, including sensory neurons and developing or stressed motor neurons (Ibanez and Simi, 2012, Xie et al., 2003).

We outline the protocol for injection of these fluorescently tagged probes into: (1) the tibialis anterior (TA) and gastrocnemius muscles (GC) of the hindlimb, allowing labelling of both motor and sensory axons of the sciatic nerve; and (2) the footpad, allowing for the specific labelling of sensory neurons. Finally, we discuss methods for the detailed analysis of axonal transport characteristics.

## 2 Materials and methods

### 2.1 Reagents

The following reagents are required:

BL21(DE3)pLys *Escherichia coli* bacteria (Agilent Technologies, cat. no. 230134), pGEX-4T3 vector (GE Life Sciences, cat. no. 28-9545-52), isopropyl ß-d-1-thiogalactopyranoside (IPTG; Sigma Aldrich, cat. no. I5502), phosphate buffered saline (PBS; Sigma Aldrich, cat. no. P4417), Tween^®^ 20 (Sigma Aldrich, cat. no. P9416), phenylmethylsulfonyl fluoride (PMSF; Fluka-Sigma Aldrich, cat. no. 78830), benzamidine hydrochloride hydrate (Fluka-Sigma Aldrich, cat. no. 12073), glutathione-agarose (Sigma Aldrich, cat. no. G4510), human thrombin (Sigma Aldrich, cat. no. T6759), Bradford protein assay (Bio-Rad, cat. no. 500-0006), tris-(2-carboxyethyl)phosphine hydrochloride (TCEP; Thermo Scientific, cat. no. 20490), dimethyl sulfoxide (DMSO; Sigma Aldrich, cat. no. 41648), AlexaFluor555C_2_ maleimide (Life Technologies, cat. no. A-20346), AlexaFluor647 antibody labelling kit (Life Technologies, cat. no. A-20186), recombinant human BDNF (50 ng/μl in distilled water; Peprotech, cat. no. 450-02), isoflurane (National Veterinary Services, UK), 70% ethanol solution (v/v in distilled water), saline (0.9% NaCl w/v).

### 2.2 Equipment and software

The following equipment (or similar alternatives) is required: Superdex 200HR gel filtration column (GE Healthcare, cat. no. 17-1088-01), Amicon Ultra, Ultracel-30K filter device (Millipore, UFC803024), PD10 desalting column (GE Healthcare, cat. no. 17-0851-01), Amicon Ultra-0.5 filter device (Millipore, UFC505096), Nanodrop spectrophotometer (Labtech International), Zeiss LSM 780 NLO Multiphoton-prepared confocal microscope, oil immersion 40× objective (Zeiss EC PlnN 40×/1.3 Oil DIC II), oil immersion 63× objective (Zeiss Plan Apo 63×/1.4 Oil DIC II), immersion oil Immersol™ 518 F fluorescence free (cat. no. 444960-0000-000), tuned damped optical table (Smart Table ST-UT2 Series, Newport), environmental chamber (Zeiss XL multi S1 DARK LS; cat. no. 411857-9420-000), computer with microscope control and image acquisition software (Zen System 2012, cat no. 410135-1003-120), anaesthetic chamber and mask, hair clipper, cotton swabs, thermal blanket (such as a recirculating warm water or infrared heating pad), Zeiss Opmi MDU operating microscope with stand, surgical tape (3M™ Micropore™, cat. no. 1530S-1), scalpel (Swann Morton no. 15 disposable scalpels, cat. no. 0505), microsyringe (Hamilton, model 701 RN, 26s gauge/51 mm; cat. no. 80330), sterile suture material (PDS™ II 6-0, 45 cm length, needle 3/8 circle; Ethicon), curved forceps (John Weiss International, Jewellers Forceps, no. 7, curved, cat. no. 0101374), straight forceps (John Weiss International, Jewellers Forceps, no. 5 straight, very fine, no. 0101472), scissors, Parafilm^**®**^, microscope stage for imaging of small rodents (custom made for Zeiss LSM 780; Digital Pixel), glass coverslips (VWR International, 22 mm × 64 mm, thickness 1.0, cat. no. 631-0142).

Time-lapse images were analysed using ImageJ software for kymograph generation (http://imagej.nih.gov/ij/download.html) and Kinetic Imaging for tracking of individual endosomes (other similar motion analysis software can also be used).

### 2.3 Equipment setup

The protocol utilises an inverted confocal microscope (Supplementary Fig. 1) equipped with a 40× EC Plan-Neofluar oil immersion objective with a numerical aperture (NA) of 1.3 and a 63× Plan-Apochromat oil immersion objective with a NA of 1.4. AlexaFluor555-conjugated H_C_T was visualised using the 561 nm laser line, whilst the AlexaFluor647-conjugated p75^NTR^ antibody was visualised using the 633 nm laser line.

The microscope is controlled by a computer with appropriate software for image acquisition. Ideally, the microscope should be housed within a darkened environmental chamber with an attached heating unit (Supplementary Fig. 1), allowing all equipment to be pre-warmed to 37 °C, the mouse's body temperature to be stably maintained and outside light to be excluded. The microscope stage was replaced with a custom-made stage with a 45 × 18 mm window, covered with a glass coverslip (22 × 64 mm, thickness no. 1) (Supplementary Fig. 2).

### 2.4 Mice

Mice with a body weight over 17 g are recommended, as the sciatic nerve is easier to expose and image in larger mice. Larger mice are also easier to stably anaesthetise. For reference, the youngest age our laboratory has reproducibly imaged is 25 days (Bilsland et al., 2010).

All animal work was carried out under license from the UK Home Office in accordance with the Animals (Scientific Procedures) Act 1986 (Amended Regulations 2012).

### 2.5 Preparation of fluorescently labelled H_C_T and α-p75^NTR^

#### 2.5.1 H_C_T expression and purification

H_C_T (H_C_T^441^, residues 875–1315), fused at its amino-terminus to an improved cysteine-rich tag (FLN CCP GCC MEP) (Martin et al., 2005) and a human influenza haemagglutinin (HA) epitope (YPY DVP DYA), was expressed as a glutathione-*S*-transferase (GST) fusion protein in BL21(DE3)pLys *E. coli* using the pGEX-4T3 vector (Restani et al., 2012). This version of H_C_T has been optimised for longer shelf life and low processing by tissue proteases. The fusion protein was expressed for 4 h at 30 °C in the presence of 0.4 mM IPTG. Bacteria were pelleted, washed with PBS containing 0.05% Tween 20 (PBST) and resuspended in resuspension buffer (PBST containing 2 mM EDTA, 0.1% 2-mercaptoethanol, 0.5 mM PMSF and 1 mM benzamidine). The suspension was then subjected to two cycles of snap freezing and thawing and sonicated three times at maximum power for 15 s. The resulting lysate was ultracentrifuged at 28,000 rpm for 30 min The cleared bacterial extract was incubated with glutathione-agarose beads for 2 h at 4 °C. The bound GST-fusion protein was then washed with PBST, incubated with 50 mM tris(hydroxymethyl)aminomethane–HCl (Tris–HCl) pH 7.4, 2 mM ATP, 10 mM MgSO_4_ for 10 min at 37 °C followed by PBST containing 0.5 M NaCl. The tagged H_C_T were released by thrombin cleavage (20 NIH units of human thrombin in 1 ml of 50 mM Tris–HCl pH 8.0, 150 mM NaCl, 0.1% 2-mercaptoethanol) for 30 min at 25 °C, and further eluted in the same buffer without thrombin containing 500 mM NaCl. The pooled fractions were concentrated and purified by gel filtration on Superdex 200HR equilibrated in 20 mM HEPES-NaOH, pH 7.4, 150 mM NaCl (0.4 ml/min). Purity was assessed by SDS–PAGE and factions containing a mix of monomeric and dimeric H_C_T were pooled, concentrated using Amicon Ultracel-30K (30,000 Da MWCO) filters, aliquoted, snap frozen in liquid nitrogen and stored at −80 °C. Protein concentration was determined by Bradford assay using purified bovine serum immunoglobulins as standard.

#### 2.5.2 Fluorescent labelling of H_C_T with AlexaFluor555

1.5 mg H_C_T protein was diluted in labelling buffer (10 mM HEPES-NaOH pH 7.4, 250 mM NaCl) containing 1.5 mM of the reducing agent TCEP and incubated for 30 min at 4 °C. 1 mg AlexaFluor555 C_2_ maleimide was reconstituted in 30 μl DMSO, and added under stirring to the reduced H_C_T (11 μl per 100 μl of reaction mix). The resulting mixture was incubated at 4 °C overnight in the dark with constant agitation. The labelling reaction was stopped by addition of 4 mM reduced glutathione in Tris–HCl (pH 8.0) for 30 min. The labelled protein was isolated from free dye using a PD10 desalting column, equilibrated with ice-cold PBS, according to the manufacturer's instructions and then dialysed over two days against ice-cold dialysis buffer (10 mM HEPES-NaOH, 100 mM NaCl, pH 7.4) at 4 °C in the dark. Finally, the labelled protein was concentrated using an Amicon Ultra-0.5 filter device according to the manufacturer's instructions. The maximum volume that can be injected into the TA or GC muscle or footpad is approximately 2 μl; therefore ensure that the concentration of the probe is ≥6.5 μg/μl. AlexaFluor555-conjugated H_C_T was aliquoted, snap frozen and stored at −80 °C until use.

#### 2.5.3 Fluorescent labelling of the p75^NTR^ antibody with AlexaFluor647

The antibody used in this protocol (5410) was raised against the extracellular domain of p75^NTR^ and has been previously described (Deinhardt et al., 2007). In order to achieve good labelling, the p75^NTR^ antibody was first dialysed in PBS at 4 °C under stirring for 48 h, to remove any contaminating ammonium ions or primary amines. Next, the antibody was concentrated to 1 mg/ml using an Amicon Ultra-0.5 filter device according to the manufacturer's instructions, as this is the optimal concentration for efficient labelling. The antibody was then labelled using the AlexaFluor647 monoclonal antibody labelling kit according to the manufacturer's instructions. The concentration of the labelled antibody was determined using a Nanodrop spectrophotometer and the antibody then stored at 4 °C in the dark for up to 2 months.

### 2.6 Injection of fluorescently labelled probes

Good labelling of the sciatic nerve was achieved using 13 μg of fluorescently labelled H_C_T and 50 ng BDNF or 2 μg fluorescently labelled α-p75^NTR^ and 50 ng BDNF. BDNF was included as it enhances the uptake of the fluorescently labelled probes into the nerve (Roux et al., 2006). Injection of H_C_T into the TA and GC muscles labelled both motor and sensory neurons within the sciatic nerve, whilst injection into the footpad allowed selective labelling of sensory neurons. The use of α-p75^NTR^ allows the labelling of endosomes within p75^NTR^-expressing cells, including sensory neurons and developing or stressed motor neurons (Ibanez and Simi, 2012, Xie et al., 2003). Intramuscular (i.m.) and footpad injections were performed in different animals.

#### 2.6.1 Injection of fluorescently labelled probes into the TA and GC muscles

The fluorescently labelled H_C_T (or α-p75^NTR^) and BDNF were mixed just before inducing anaesthesia and kept on ice until the time of injection. The mouse was anaesthetised using isoflurane and fur was removed from the right hind limb using clippers. The animal was placed on a thermal blanket to minimise heat loss during surgery. Skin in the operating area was then disinfected using 70% ethanol and the right hind limb was immobilised by taping the paw to the operating table. Small incisions (∼0.5 mm) were made through the skin to expose the TA and GC muscles (Fig. 1A). The Hamilton syringe was loaded with fluorescently labelled probe/BDNF and inserted into the centre of the TA muscle (at an angle of 30 degrees from the plane of the table), until the eye of the needle was just embedded in the muscle. 6.5 μg of AlexaFluor555-conjugated H_C_T (or 1 μg AlexaFluor647-conjugated α-p75^NTR^) and 25 ng BDNF were slowly co-injected (Fig. 1B). The needle was left in place for 10 s before being slowly removed, in order to minimise leakage of the probe. This process was then repeated for the GC muscle, inserting the needle into the belly of the muscle (1–2 mm deeper than for the TA muscle), at an angle of 60 degrees to the plane of the table (Fig. 1C). The skin was then sutured closed and the mouse returned to its cage to recover.

#### 2.6.2 Injection of fluorescently labelled probes into the footpad

13 μg AlexaFluor555-conjugated H_C_T (or 2 μg AlexaFluor647-conjugated p75^NTR^ antibody) and 50 ng BDNF were mixed just before inducing anaesthesia and kept on ice until the time of injection. The mouse was anaesthetised using isoflurane and placed on a thermal blanket to minimise heat loss during surgery. The fluorescently labelled probe and BDNF were slowly co-injected into the footpad and the needle left in place for 10 s before being removed. The use of non-topical non-steroidal anti-inflammatory drugs is advised for pain management when injecting into the footpad, in combination with close monitoring during recovery.

### 2.7 Surgical exposure of the sciatic nerve at the mid-thigh level

Axonal transport was assessed in the sciatic nerve 4 h after injection of the fluorescently labelled probes, to allow sufficient time for uptake of the probe into the nerve and initiation of axonal transport. From our experience, labelling of the sciatic nerve persists up to at least 24 h after injection (Supplementary Fig. 3).

The mouse was re-anaesthetised, placed on a thermal blanket and an incision made from the hip to the knee of the right hind leg using a scalpel (Fig. 2A). Using scissors, the skin on both sides of the incision was cut away (Fig. 2B) and the overlying muscle removed in order to expose the sciatic nerve in the mid-thigh region (Fig. 2B, arrow). The surrounding muscles and connective tissue were then carefully cut away from either side of the nerve (Fig. 2C), avoiding any blood vessels that, if cut, would flood the preparation with blood making imaging of the nerve very difficult. The sciatic nerve was then separated from the underlying tissue by inserting a pair of curved tweezers underneath the nerve and carefully teasing it free (Fig. 2D). Finally, a small piece of Parafilm^®^ was inserted beneath the nerve to separate it from the underlying tissue (Fig. 2E) and allow for easier location and imaging of the nerve under the microscope.

### 2.8 Live confocal imaging of the exposed sciatic nerve

The anaesthetised mouse was transferred to the microscope stage (Fig. 3A) within the environmental chamber, both of which were pre-warmed to 37 °C. A small drop of saline was applied to the glass coverslip to ensure the sciatic nerve did not dry out during imaging. The nerve was then carefully positioned directly above the objective, ensuring it made good contact with the glass coverslip (Fig. 3B). The sciatic nerve was located using the 40× objective and the 63× objective was used to visualise labelled axons (Fig. 3C and Supplementary Video 1). A pinhole of 1.3 Airy Units (1.2 μm section) was used and laser power was kept below 2% to avoid phototoxicity. An area containing 1–3 labelled axons was selected and imaged at 100× using digital magnification (Fig. 3D). The image frame was approximately 60 μm by 15 μm and the optimal number of pixels was chosen that allowed for the best resolution to be obtained (700 × 700 pixel). Line averaging of 8 was used to reduce noise. A time series was acquired, collecting images every 2–6 s (pixel dwell times between 0.6 and 0.8 μs) for up to 500 frames (Supplementary Video 2). At least three time series were collected per experiment, imaging different areas of the nerve each time. After imaging, the mouse was immediately euthanised.

### 2.9 Analysis of axonal transport movies

Time series images were converted into .avi files for analysis using Kinetic Imaging software. The speed of axonal transport was analysed by determining the distance moved by each endosome between consecutive frames. Endosomes were only tracked if they crossed the entire width of the image frame. The speeds of individual steps are presented as a frequency histogram using a 0.2 μm/s binning interval (Fig. 4A, C–E). Error bars represent standard error of the mean.

Alternatively, ImageJ can be used to create kymographs from time series images (Fig. 4B) and the average speed of individual cargoes determined as the distance moved within the given observation time (for a detailed description of kymograph generation and analysis see De Vos and Sheetz, 2007).

In order to determine statistical significance of axonal transport data, the average speed is calculated by taking the mean of the mean speeds within individual tracks for each biological replicate and a *t*-test (2 experimental groups) or ANOVA (> 2 experimental groups) is performed.

## 3 Results and discussion

We have established a live imaging technique that allows visualisation of the axonal transport of individual endosomes *in vivo* in the mouse sciatic nerve and differentiation of axonal transport in motor and sensory neurons. The entire protocol can be undertaken within a period of 6 h, allowing the rapid collection of robust *in vivo* data.

One limitation of this method is the restricted temporal resolution. This is controlled by the size of the observation field, and is a problem inherent with the use of confocal microscopy. However, the acquisition of frames every 2–4 s, as described in this protocol, allows individual endosomes to be resolved whilst still enabling the easy tracking of a particular endosome from one frame to the next. The temporal resolution is therefore sufficient to gain high quality data on *in vivo* endosome dynamics. We also observed that axonal transport speeds recorded *in vivo* are significantly faster than those recorded *in vitro* and show a lesser degree of variability (Fig. 4A), demonstrating the robustness of our protocol in generating physiologically-relevant and reliable data.

In the protocol described above, we analysed the speed of individual steps taken by single endosomes and represented this data as speed distribution profiles (Fig. 4A, C–E). This approach allows a vast amount of data to be presented in a format that is easy to interpret and that can be tested for statistical significance. Our analysis method also allows the contribution of pauses to be considered. However, it is also possible to present and analyse axonal transport data in a variety of other ways. Analysis of parameters such as the frequency of motile cargoes (Bilsland et al., 2010, De Vos and Sheetz, 2007), percentage of stationary cargoes and stop length (Marinkovic et al., 2012), average speed (De Vos and Sheetz, 2007, Marinkovic et al., 2012, Zhang et al., 2010) and net displacement (De Vos and Sheetz, 2007) have been previously described in detail and could easily be applied to the data generated from our *in vivo* imaging approach.

The live imaging technique described above enables the *in vivo* monitoring of alterations in axonal transport in mouse models of neurological disease. Such analysis has been performed previously in our laboratory for both the SOD1^G93A^ mouse model of ALS (Bilsland et al., 2010) and the AR100 mouse model of spinal bulbar muscular atrophy (Malik et al., 2011). Deficits in axonal transport were found selectively in motor neurons at a presymptomatic stage in the SOD1^G93A^ mouse, and these defects worsened at an early symptomatic stage (Fig. 4C and D; Bilsland et al., 2010). These studies demonstrate the usefulness of our *in vivo* imaging protocol to analyse axonal transport at different disease stages and thereby gain insights into the role that deficits in axonal transport may play in disease pathogenesis. These results also highlight the ability of our protocol to differentiate motor and sensory neurons (Fig. 4E) and to determine their individual roles in disease pathogenesis (Fig. 4C and D; Bilsland et al., 2010). There is still much debate over the role of axonal transport defects in neurodegeneration, and the ability to image axonal transport *in vivo* at different stages of disease progression in both motor and sensory neurons will be crucial to solve this controversy. This protocol can also be used to investigate the effect of pharmacological treatments on axonal transport *in vivo*, in order to identify potential novel therapeutics for neurological diseases in which axonal transport defects have been implicated.

Finally this protocol allows the detailed study of cargo-specific mechanisms of axonal transport *in vivo*. We foresee that this method could easily be adapted to monitor the axonal transport of RNA (using dyes such as SYTO RNA Select from Life Technologies), fluorescently labelled growth factors (for example, quantum-dot conjugated BDNF; Xie et al., 2012), autophagosomes (using LC3-GFP mice; Mizushima and Kuma, 2008) and lysosomes (using Lysotracker™ or other lysosome-specific dyes; Ho et al., 2014). Studying the axonal transport of RNA in motor neurons *in vivo* will be particularly interesting in ALS, where mutations in RNA binding proteins implicated in the regulation of RNA axonal transport have been found in patients (Alami et al., 2014, Gitcho et al., 2008, Kabashi et al., 2008, Kwiatkowski et al., 2009, Sreedharan et al., 2008, Vance et al., 2009).

## 4 Conclusions

This new method for imaging axonal transport in the sciatic nerve of live, anaesthetised mice allows the rapid collection of reliable *in vivo* data that will further our understanding of axonal transport and its role in neurological diseases.

## Author contributions

KLG and BK optimised and performed the surgery. KLG, BK and JNS carried out the *in vivo* imaging; KLG tracked axonal signalling endosomes, performed the analysis and assembled the figures; GS expressed and purified H_C_T; KLG and GS wrote the manuscript. All authors amended the manuscript and approved the submitted version.

## Conflict of interest statement

The authors declare no competing financial interests.

## Figures and Tables

**Figure:**
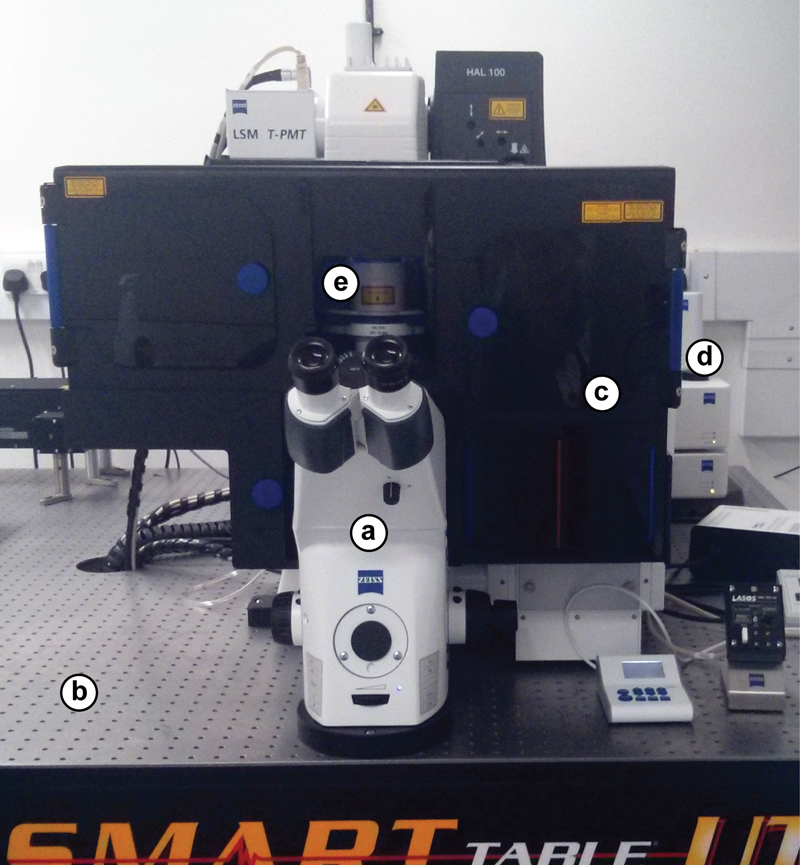


**Figure:**
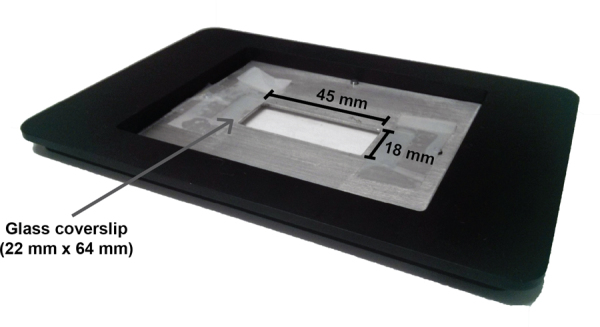


**Figure:**
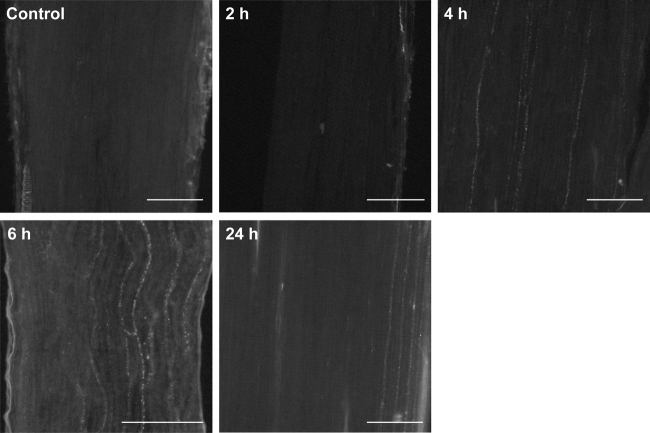


**Fig. 1:**
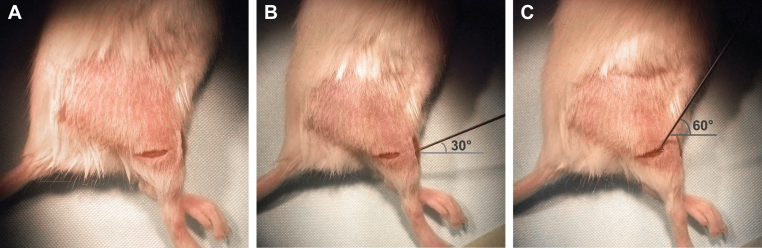
*Intramuscular injection of fluorescently labelled probes into the tibialis anterior and gastrocnemius muscles.* (A) Incisions are made through the skin above the tibialis anterior (TA) and gastrocnemius (GC) muscles in one hind leg to expose the muscles. (B) Positioning of the needle for injection of the fluorescently labelled probe into the exposed TA muscle. (C) Positioning of the needle for injection of the fluorescently labelled probe into the exposed GC muscle.

**Fig. 2:**
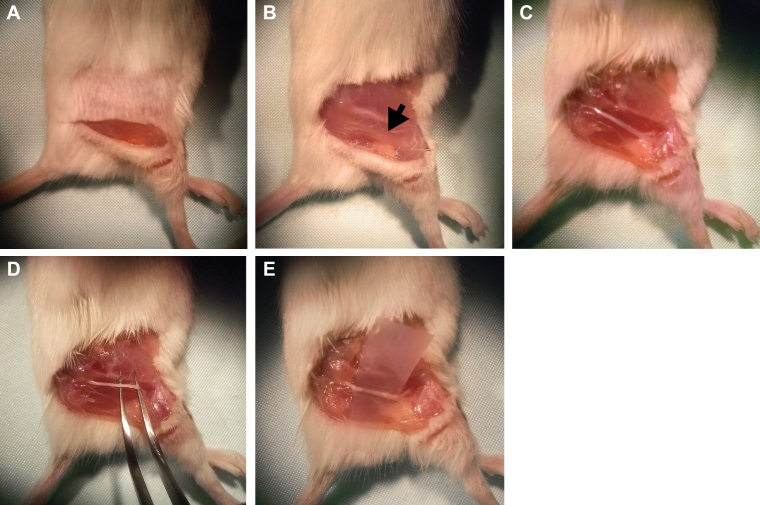
*Surgical exposure of the sciatic nerve for in vivo imaging.* (A) An incision is made through the skin between the knee and the hip joint to expose the underlying muscle and nerves. (B) The skin around the operating field is removed and the sciatic nerve (black arrow) is visible underneath the muscle, below the femur. (C) Muscle is carefully removed to reveal the underlying sciatic nerve. (D) The sciatic nerve is separated from the surrounding tissue using a pair of curved forceps. (E) A small piece of Parafilm^®^ is inserted beneath the sciatic nerve to separate it from underlying tissues, thus facilitating the imaging of the nerve.

**Fig. 3:**
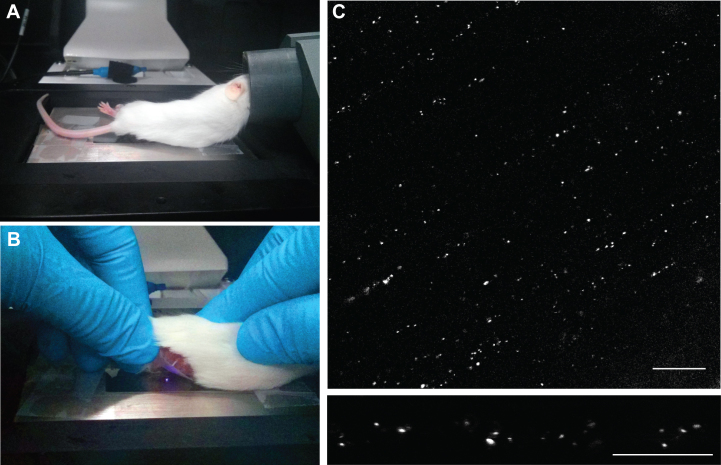
*In vivo imaging of the exposed sciatic nerve using confocal microscopy.* (A) Correct positioning of the anaesthetised mouse on the microscope stage. (B) The exposed sciatic nerve is carefully placed on the glass coverslip, directly above the microscope objective. (C) Raw image of the sciatic nerve using the 63× objective, showing labelled endosomes within axons. Scale bar, 20 μm. (D) Imaging of a collection of axons at 100× allows sufficient time and spatial resolution for individual endosomes to be tracked. Scale bar, 10 μm.

**Fig. 4:**
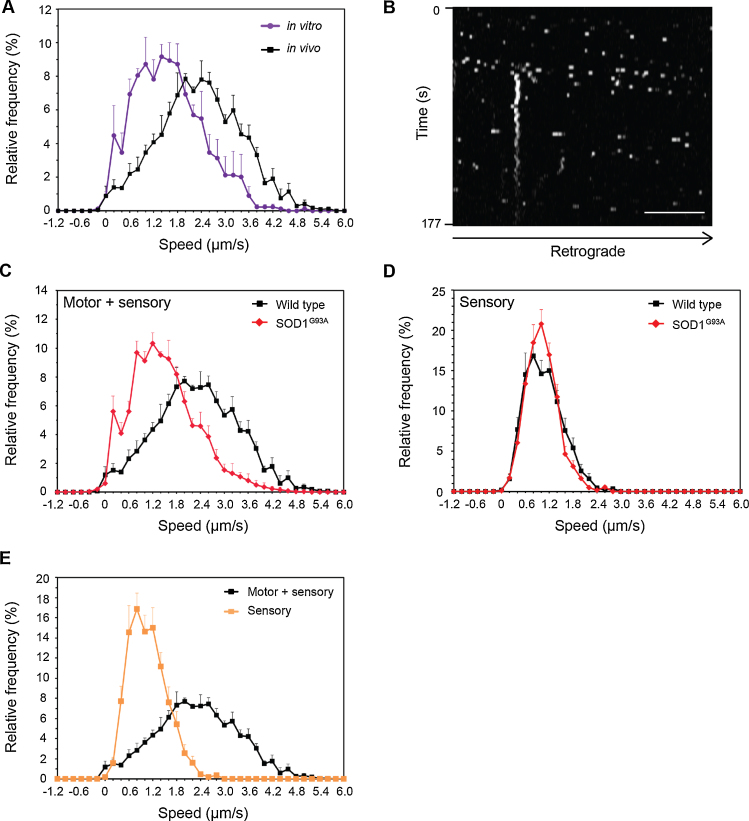
*Analysis of axonal transport.* (A) A comparison of *in vitro* and *in vivo* axonal transport speed distribution curves of H_C_T-labelled signalling endosomes. The speed profiles reveal that H_C_T-labelled endosomes move in the intact sciatic nerve at significantly faster speeds than those observed *in vitro* in cultured primary motor neurons. (B) Typical kymograph generated from a wild type *in vivo* confocal time series. Scale bar, 10 μm. (C and D) Deficits in axonal retrograde transport of signalling endosomes are present in motor but not sensory neurons of the SOD1^G93A^ mouse model of amyotrophic lateral sclerosis (ALS). (C) AlexaFluor555-conjugated H_C_T was injected i.m. and retrograde transport in single axons of the sciatic nerve was assessed in 73-day old wild type and SOD1^G93A^ mice. SOD1^G93A^ mice displayed a significant impairment in retrograde transport compared to wild type controls (*p* = 0.001); wild type: 161 carriers, *n* (animals) = 4; SOD1^G93A^: 185 carriers, *n* = 5). (D) AlexaFluor555-conjugated H_C_T was injected into the footpad of wild type and SOD1^G93A^ mice (73 d; pre-symptomatic stage) to allow selective labelling of sensory neurons in the sciatic nerve. The speed profiles reveal that there is no defect in axonal transport in sensory neurons of SOD1^G93A^ mice, indicating that the deficits seen in the sciatic nerve after i.m. injection of H_C_T (C) must be due to disrupted axonal transport in motor axons (data adapted from Bilsland et al., 2010). (E) A comparison of *in vivo* axonal transport speeds in the sciatic nerve of wild type mice after H_C_T is injected i.m. (motor and sensory axons labelled) or into the footpad (only sensory axons labelled) (footpad data adapted from Bilsland et al., 2010).
