# Supplementary figures and images for: In vivo imaging of axonal transport in murine motor and sensory neurons
Source: J Neurosci Methods. 2016 Jan 15;257:26–33. doi: 10.1016/j.jneumeth.2015.09.018 (PMC4666412; doi:10.1016/j.jneumeth.2015.09.018)

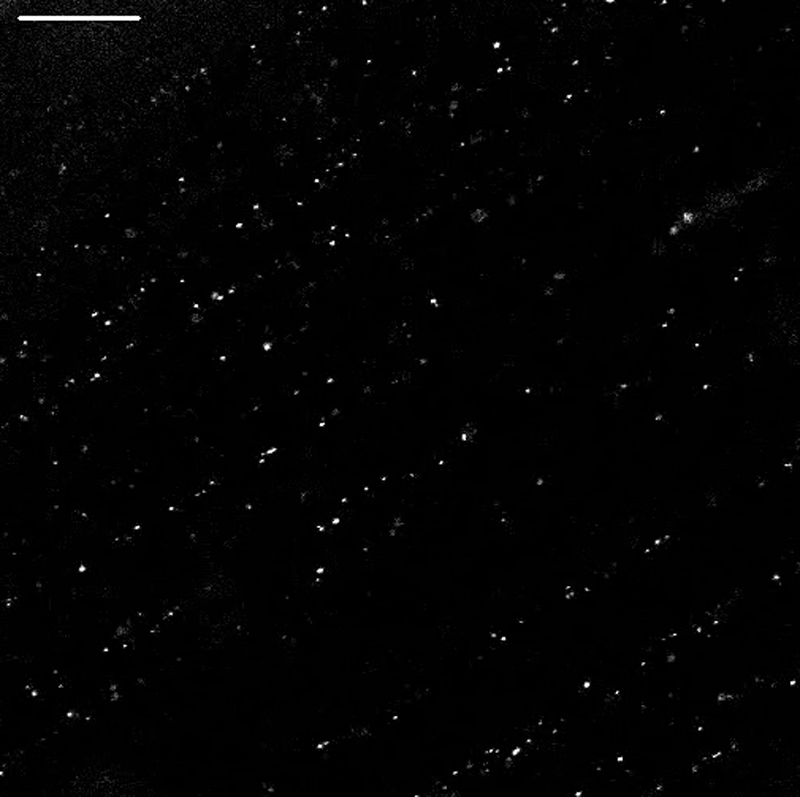

Supplement: Supplementary file 1 [file mmc4.jpg]

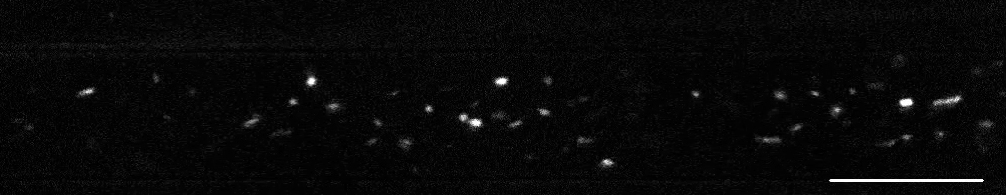

Supplement: Supplementary file 2 [file mmc5.jpg]
